# Exploring relationships between pre-service teachers’ self-efficacy for writing and instruction provided in simulated elementary writing conferences

**DOI:** 10.3389/fpsyg.2023.1214086

**Published:** 2023-09-27

**Authors:** Amy Gillespie Rouse, Murphy K. Young, Diane Gifford

**Affiliations:** Department of Teaching and Learning, Southern Methodist University, Dallas, TX, United States

**Keywords:** writing, elementary education, simulation, self-efficacy, motivation, teacher preparation

## Abstract

Practice-based opportunities, like teaching simulations, are becoming more prevalent in teacher preparation programs. We sought to examine the instructional moves of 5 pre-service teachers during a simulated elementary writing conference using Mursion technology, a mixed-reality simulation (MRS) that emulates a classroom environment with student avatars. We examined both participants’ self-efficacy and their instructional moves during MRS writing conferences. To better understand pre-service teachers’ learning, we also examined reflections they wrote about their MRS experience. Results showed that pre-service teachers spent much of their time (31.7%) managing the environment (e.g., setting expectations, addressing student behavior) during MRS writing conferences, followed by nearly one-fourth of their time (24.2%) instructing students on their writing pieces (e.g., adding details, revising, editing), with high levels of teacher talk compared to student talk. Participants’ self-efficacy for writing, for teaching writing elements, and for writing instruction were not clearly related to their instructional moves during the MRS experience. However, participants’ reflections suggest that pre-service teachers felt the experience gave them the opportunity to practice making in-the-moment decisions and learn from their peers in a way that may allow them to have a more accurate understanding of their abilities to teach writing. Implications from these findings related to teacher self-efficacy, motivation, and teacher preparation programs are presented.

## Introduction

An examination of literacy courses in U.S. teacher preparation programs shows an emphasis on reading over writing, even when courses include the word *writing* in their titles and descriptions (Myers et al., 2016; Brenner and McQuirk, 2019). Not surprisingly then, elementary and secondary teachers in the U.S. report receiving little preparation to teach writing or to help students use writing to support their learning (Kiuhara et al., 2009; Gilbert and Graham, 2010; Ray et al., 2016; Gillespie Rouse et al., 2021). K-12 students’ writing performance reflects this lack of teacher preparation, as U.S. students continue to perform poorly on national assessments of writing and their scores have remained relatively unchanged for years (Salahu-Din et al., 2008; National Center for Education Statistics, 2012; White et al., 2015).

There is a clear need to address the lack of preparation to teach writing in the U.S., as teacher quality has a powerful influence on student achievement (Darling-Hammond, 2000; Myers et al., 2016). Opportunities for pre-service teachers to learn how to provide effective writing instruction in teacher preparation programs can help increase their writing instructional skills as well as their self-efficacy for teaching writing (Grisham and Wolsey, 2011). Additionally, when pre-service teachers can apply their learning in authentic contexts, they feel more prepared entering the classroom (Ronfeldt et al., 2014). However, research on best practices for teacher education on writing methods is limited (Myers et al., 2016; Sanders et al., 2020). Thus, we designed a practice-based rehearsal for pre-service teachers to apply writing instructional moves taught in our literacy methods course. We aimed to add to the literature on practice-based opportunities for pre-service teachers, specifically in writing, and to examine if participants’ instructional moves during the rehearsals were related to their self-efficacy.

## Review of literature

### Practice-based teaching opportunities

Teacher preparation programs are becoming increasingly more practice-based (Cohen et al., 2020). Practice-based opportunities, sometimes referred to as approximations of practice (Grossman et al., 2009), encompass a variety of instructional techniques that can occur within coursework and field experiences. Forzani (2014) defines this approach to teacher education as involving training that focuses “novices’ learning more directly on the work of teaching,” (p. 357). Approximations provide pre-service teachers the opportunity to rehearse certain skills, such as in-the-moment decision-making and application of evidence-based teaching practices, before they enter the classroom (Grossman et al., 2009). By participating in approximations of teaching practices, pre-service teachers can rehearse, pause, get feedback from peers and instructors, and reflect on practice in ways that are not possible in actual classrooms (Grossman et al., 2009; Lampert et al., 2013; Benedict et al., 2016). During these opportunities, embedded coaching, feedback, and reflection support pre-service teachers’ understanding and implementation of instruction, helping to bridge coursework with field experiences (Darling-Hammond, 2006).

One approximation of practice, the teaching simulation, has become more frequently integrated into teacher preparation courses (Ronfeldt, 2021). Teaching simulations allow pre-service teachers to rehearse providing instruction to “students” enacted through technology (i.e., student avatars) or with live actors (e.g., Kane, 2020). Mursion is one type of simulation technology that uses mixed-reality software to emulate a small group of students within a classroom setting (Landon-Hays et al., 2020). Mursion is deemed a “mixed reality” simulation (MRS) because it has both human and technological components that interact to provide authentic teaching experiences (Hartle and Kaczorowski, 2019). Users instruct in a virtual classroom environment, but student avatars respond in real-time because they are controlled through live actors (Cohen et al., 2020). With the ability to pause and restart instruction, peers and teacher educators can observe, provide feedback, and collaborate to work through obstacles that may arise during lessons implemented with Mursion (Dieker et al., 2014).

Researchers are still exploring ways simulations, like Mursion, are used within the context of teacher preparation. In a recent scoping review of physical simulation and MRS for pre-service teachers (Ade-Ojo et al., 2022), researchers found that although the research base was small, simulations were a promising tool for increasing pre-service teachers’ confidence, communication, management skills, and self-efficacy. In terms of content area instruction, MRS research has been concentrated largely in math (e.g., Grant and Ferguson, 2021). We identified little research (Young and Gillespie Rouse, n.d.) using MRS for teacher preparation in literacy, with most of these studies focused on reading (e.g., Ely et al., 2018), and no research yet focused specifically on writing instruction for pre-service teachers using MRS.

### Elementary writing conferences

In the context of our university literacy course, we designed a MRS with a focus on enacting elementary writing conferences. We felt this was an important opportunity for pre-service teachers because effective writing instruction is critical in the elementary grades (Graham et al., 2012). Beginning in elementary school, students typically learn to carry out the writing process (i.e., planning, drafting, revising, editing, and publishing) through iterative cycles of writing, sharing, and getting feedback on their work (Graves, 1983; Ray and Cleaveland, 2004). A critical component of this process is the writing conference, during which students get feedback from teachers (or peers) and leave with concrete next steps for their writing (Green and Steber, 2021). The writing conference provides a context for individualized support and instruction in writing skills, but perhaps more importantly, supports the development of a student’s writing craft and their confidence in their writing abilities (Anderson, 2001; Hale, 2017; Myroup, 2020).

During a writing conference, teachers’ instructional moves can affect not only the quality of students’ writing but also students’ self-regulation of the writing process and their beliefs that they can reach writing goals (Helsel et al., 2022). Anderson (2018) suggested that in successful writing conference teachers should seek to understand how a student feels about their writing process, assess their current strengths and needs within the writing piece, and focus on one writing skill to teach the student. Over time, writing conferences should support the development of the student as a writer, as opposed to making corrections to each of the student’s writing pieces and they should always operate from a student-centered and individualized approach (Anderson, 2001, 2018; Helsel et al., 2022).

In this way, effective writing conferences require preparation as well as on-the-fly decision-making based on listening and responding to a student’s needs and contributions during the conference. Accomplishing all of these tasks within a relatively short timeframe (conferences are typically brief and individualized for each student) is difficult to negotiate, even for experienced teachers (Lipson et al., 2000).

## Theoretical framework

For this study, we chose to examine how pre-service teachers’ self-efficacy for writing and writing instruction might guide their instructional moves within a simulation experience. Stated simply, self-efficacy is related to an individual’s judgments of how well they can carry out a course of action to accomplish a task (Bandura, 1982). We situated our work within Bandura’s (1986) social cognitive theory, using the lens that self-perceptions, or self-efficacy beliefs, have strong influences on behavior.

In applying Bandura’s theory within the context of writing instruction, Pajares (2003) posited that writing self-efficacy could be further parsed into self-efficacy of students’ writing skills, their confidence in completing writing tasks, and their perceptions of their own proficiency in a language arts course. Hodges et al. (2021) applied this framework to understanding pre-service teachers’ self-efficacy for writing, identifying four main sources contributing to writing self-efficacy development for pre-service teachers: past experiences with writing, instruction from teachers and peers, understanding different social perspectives of writing, and personal beliefs about writing.

Higher teacher self-efficacy can have positive impacts on both teachers and students (Zee and Koomen, 2016). Teachers with higher self-efficacy tend to have higher rates of persistence and resilience and are more likely to continue in the classroom (Yost, 2006; Pedota, 2015). In the area of writing specifically, studies showed that teachers with higher self-efficacy for writing provided better writing instruction to their students and had students with higher writing performance (De Smedt et al., 2016; Graham et al., 2022).

### Self-efficacy and motivation

Self-efficacy is related to motivation because self-efficacy beliefs influence which challenges an individual undertakes, how much effort they exert, how long they persevere when encountering obstacles or failures, and whether they view failures as impetus to continue or as reason to stop their efforts (Bandura, 2001). Motivation and self-efficacy increase when individuals perceive they are performing well or becoming more competent (Schunk, 1995).

Research shows that a teachers’ motivational beliefs, like their self-efficacy beliefs, are related to students’ performance as well as to teachers’ commitment to the profession (Watt and Richardson, 2012; Lauermann et al., 2017). Teachers’ motivational beliefs have also been shown to influence students’ own motivation (Richardson and Watt, 2010), engagement (Lauermann and Berger, 2021) and interest in what is being taught (Lazarides et al., 2023). Teachers with greater self-efficacy may also be more motivated to try new teaching strategies, introduce more challenging activities to their students, promote a more positive classroom environment, and address the needs of students who are struggling (Schunk, 1995).

## Research questions

We chose to focus our MRS on writing conferences because they offer critical opportunities for providing writing instruction and individualized support but are difficult to enact. We wanted pre-service teachers to practice carrying out writing conferences using the knowledge gained from our early literacy course, using tools (e.g., checklists, student writing) they would later use in their own classrooms, and problem-solving within their community (i.e., peers in class) to provide effective instruction. We chose MRS, as the use of simulation in teacher preparation can provide novice teachers with a safe and controlled environment to try out new skills and strategies (Dieker et al., 2014). Beyond their instructional benefits, practice-based opportunities, like Mursion, may also be an avenue for supporting pre-service teachers’ self-efficacy and motivation (e.g., Bautista and Boone, 2015; Gundel et al., 2019; Öner and Yaman, 2020; Bondie et al., 2023).

Three research questions guided our investigation:

1. What instructional moves do pre-service teachers make during MRS elementary writing conferences?
2. How do pre-service teachers’ instructional moves during MRS elementary writing conferences vary based on their self-efficacy for writing and writing instruction? and
3. How do pre-service teachers reflect on their learning from the MRS?

We hypothesized that participants would apply their classroom learning but would still be impeded by management and behavior issues of avatars. We also anticipated that participants with higher self-efficacy would be motivated to provide more writing instruction, as teachers with greater self-efficacy for writing have been shown to provide more (and better) writing instruction to their students (e.g., De Smedt et al., 2016). We were less certain about participants’ reflections on the MRS but hopeful that they would be able to recognize areas of strength during the writing conference and areas in which they needed additional learning or support.

## Method

We employed a mixed methods approach in this study. After initial data analysis using quantitative methods to answer research questions 1 and 2, we added a third research question focused qualitative analysis of participants’ written reflections on their MRS experiences to provide a more nuanced understanding of their instructional moves and learnings from the MRS experience.

### Participants and setting

All students (*N* = 18) in an introductory literacy course for undergraduate education and Master’s of Education majors seeking teacher certification participated in the MRS writing conferences during the last two meetings of the course. The MRS were enacted in a teaching lab with audio/visual equipment to deliver the MRS and to capture participants’ responses.

For this study, we focus on three MRS writing conferences (*n* = 5 participants) enacted during the final course meeting (see Table 1 for participant information). We chose the final three MRS sessions for three reasons. First, they included each planned MRS scenario (MRS 1: a confident student who does not want to change their writing, MRS 2: a student distracted by off-task classmates and unable to respond to feedback, and MRS 3: a less-confident student who takes constructive feedback as criticism). Second, these sessions included both undergraduate and master’s students, which we anticipated may provide a range of self-efficacy scores based on participants’ previous classroom or teaching experiences. Third, we anticipated the final three MRS groups would be the most comfortable with the MRS technology, as they had the opportunity to observe the previous groups’ MRS sessions.

**Table 1:** Participant and MRS information.

	Ethnicity	Gender	Program
MRS 1: Confident Student Scenario			
Kim	White	F	UG
Tanya	Multiple	F	UG
MRS 2: Distracted Student Scenario			
Jackie	White	F	UG
MRS 3: Less Confident Student Scenario			
Audrey	Multiple	F	M. Ed
Sophie	White	F	M. Ed

F, female; UG, Undergraduate elementary education major; M. Ed, Master’s of Education.

### Pre-intervention planning

We provided all pre-service teacher participants with an authentic fourth-grade written response to the prompt: Pretend you have been granted three wishes. Make up a story about what you would do. We encouraged participants to plan for the MRS writing conference by completing a graphic organizer they had learned about during a previous course meeting. This graphic organizer included sections labeled: Plan, Discuss, Compliment, Teach, adapted from Anderson’s (2001) guide to writing conferences. Participants planned out what to focus on during the writing conference by reviewing the student’s writing prior to the conference, identifying strengths of the writing piece, and determining next steps for the student to take in their writing, considering grade-level writing standards. After planning individually, participants worked collaboratively with a randomly assigned small group of their classmates (groups of 2–3) to plan their instruction for the writing conference for 30 min prior to the MRS.

### Pre-intervention measures

Because teachers’ motivation, beliefs, and self-efficacy have been shown to impact their instruction (e.g., Graham et al., 2001; Tschannen-Moran and Johnson, 2011; Troia et al., 2012), we had participants complete the Preservice Teacher Self-Efficacy for Writing Inventory (PTSWI; Hodges et al., 2021) prior to completing the MRS writing conferences. In addition to questions about demographic and background information (e.g., gender, ethnicity, pre-service coursework focused on writing instruction), the PTSWI includes items to measure pre-service teachers’ beliefs and self-efficacy for: writing (*n* = 10 items); teaching particular aspects, or elements, of writing (*n* = 15 items); and writing instruction more broadly (*n* = 13 items) (Hodges et al., 2021). A 5-point scale was used for each item (i.e., strongly disagree, disagree, neither disagree or agree, agree, strongly agree). For example, in section 1 (i.e., self-efficacy for writing), participants responded on a scale from strongly disagree to strongly agree to items such as, “I feel confident in my overall writing abilities” and “The majority of time I spend writing is for enjoyment.” In Section 2 (i.e., self-efficacy for teaching writing elements), participants responded about their confidence in teaching particular writing elements and the writing process, rating (from strongly disagree to strongly agree) statements such as, “As a result of my teacher preparation program, I feel confident in my ability to teach paragraph structure” and “As a result of my teacher preparation program, I feel confident in my ability to teach grammatical conventions.” In section 3 (i.e., self-efficacy for writing instruction), participants rated responses, from strongly disagree to strongly agree, such as “Writing is an important skill to teach students” and “Teachers who have more positive beliefs about writing can more effectively teach writing.”

### Intervention

**Figure 1:**
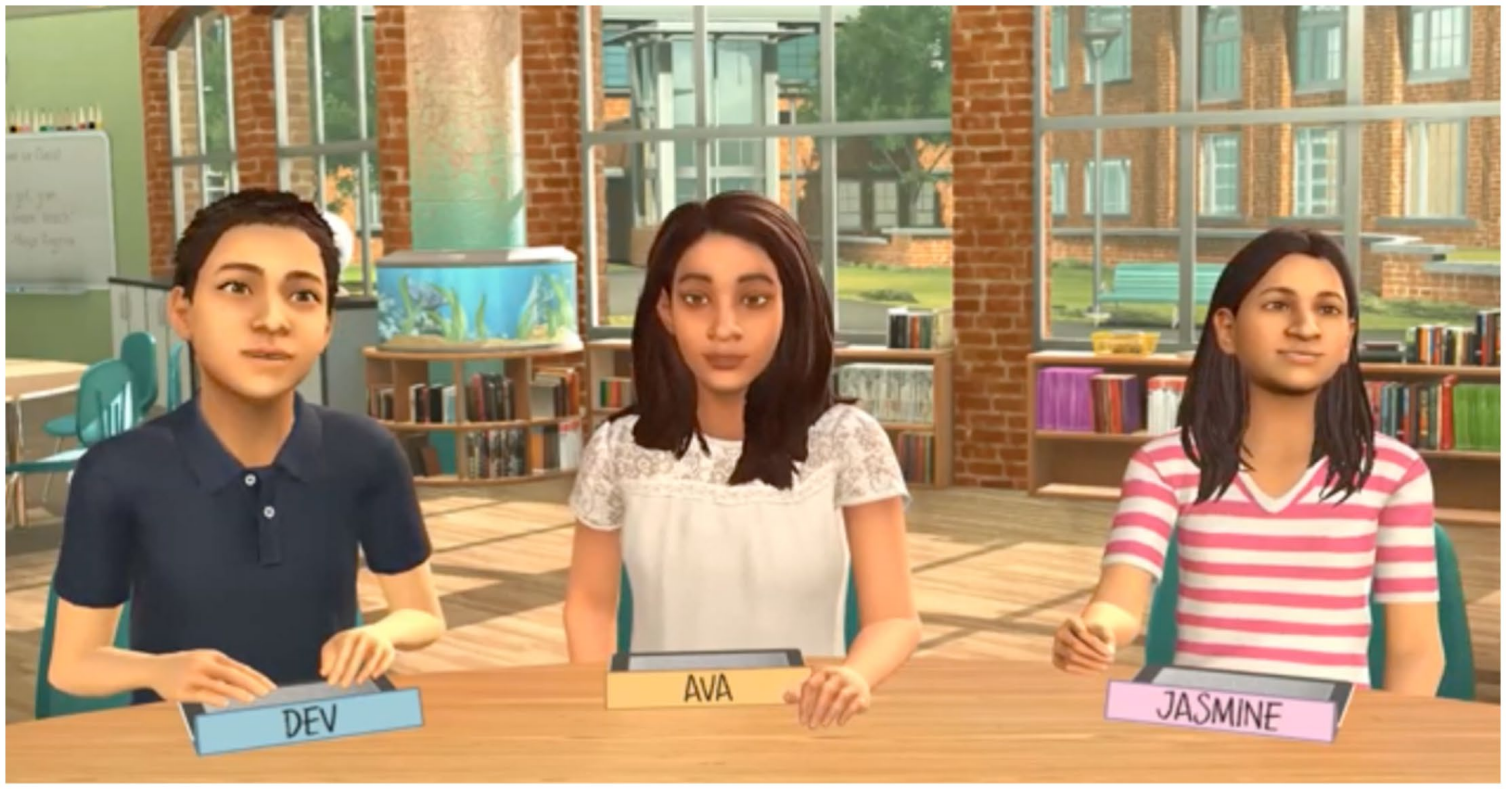
Student avatars in MRS writing conferences (screen capture from study dataset, with participant image removed).

We created three MRS experiences with elementary student avatars (see Figure 1). Each MRS represented a scenario teachers may encounter in their future classrooms during writing conferences. Participants had been introduced to Mursion software used for the MRS during an introductory activity on a different topic. We randomly assigned avatars to participants the day of the MRS, informing participants which avatar they would be working with immediately prior to the MRS beginning. These decisions ensured participants would have some familiarity with the student avatars prior to working with them but would develop a lesson plan that could be applied to any of the avatars.

Only a single participant could engage at a time with the avatars. Each small group chose a participant to begin the MRS. During the MRS, each participant sat at a table in front of a group of three avatars on a large screen. Participants engaged with all three avatars but only focused instruction on their previously assigned focal avatar. Thus, they had to instruct a focal avatar but provide some direction for the other two avatars at times. Avatars responded in real-time to participants’ instruction, simulating the actions and responses of fourth-grade students.

Participants could pause, seek feedback or assistance from their small group, switch with a peer from their group, and restart the MRS as needed. Other class participants observed the MRS silently during the enactment. In MRS 1 and 3, participants switched who was instructing, so two participants’ instructional moves and utterances were analyzed. In MRS 2, only one participant interacted with the avatars. MRS sessions lasted, on average, 6 min (*SD* = 1.11 min).

After the MRS experiences, participants completed written reflections answering the following: (1) In what ways did your team adapt plans for the writing conference while in the simulation?; (2) Explain how the following factors influenced any adaptations made to your plan during the simulation: student avatars, your peer group, anything else?; and (3) Explain how this experience might impact your planning and instruction for future writing conferences. Consider what you might do the same and/or differently.

### Data segmenting and coding

#### MRS sessions

The embedded zoom transcription software used for recording MRS sessions segmented talk into timestamped utterances based on pauses in individual speakers’ talk and speaker changes (i.e., between avatar and participants). Together, the first two authors read through the transcripts multiple times and met to confirm that the transcription software had correctly segmented utterances. There were some instances in which participants paused in the middle of a sentence or idea (we grouped these together even though the software segmented them) or when participants continued to discuss the same idea over several separated utterances (we grouped these together even though the software initially separated them). These data segmenting decisions were made so we did not overrepresent instructional codes in our analyses. Other times, participants discussed several different ideas in the same utterance, so we segmented these. Although we coded only participant talk, we left student avatar talk in each transcript to provide context when making coding decisions. Before coding, we also removed non-relevant talk. For example, participants had to initiate the MRS by saying “Begin classroom” and typically started with a greeting, such as “Hi! How are you doing today?” After segmenting all transcripts, the third author confirmed agreement at 100% for all three transcripts.

We developed a coding scheme grounded within our larger dataset (*N* = 18; Corbin and Strauss, 1990). Through iterative rounds of reviewing videos from three MRS sessions not included in the sample for this study, we conceptualized utterances pre-service teacher participants made during each MRS writing conference. We used participants’ talk, or utterances, as a proxy for the instructional moves they made during the simulated writing conferences. Our coding scheme (see Supplementary files) went through seven iterations before we felt it adequately captured the themes and patterns in participants’ utterances (Saladana, 2016), with five final codes and example utterances for each code in the final scheme: Describe, Expand, Affirm, Manage, and Instruct.

The code *Describe* captured how pre-service teacher participants described or asked questions prompting description of the student’s writing piece (e.g., What is your writing piece about?, I like how you used _______, Can you read me ____ sentence?). The code *Expand* captured how participants expanded upon the student’s writing, focusing more on the writing process and the student’s development as a writer (e.g., How did you feel when writing this?). We used the third code, *Affirm*, to categorize more general utterances that encouraged students and supported their writing (e.g., Great idea!, I love your writing!). The code *Manage* captured utterances that involved behavior management, setting expectations for behaviors expected during the writing conferences, and clarifying directions (e.g., Please work together and read each other’s writing while I work with [focal] student, Please work quietly and stop talking to your friends). The final code *Instruct* was subdivided into two categories: (1) utterances that involved instruction on the current writing piece (e.g., You could add details here, When you go back to your desk, I want you to try ________ [concrete next steps]) (2) instruction in how the student could develop as a writer (e.g., What message do you want to convey to your reader?).

The first and second author independently coded each transcript from MRS 1, 2, and 3. To determine interrater reliability (IRR), we counted the number of coding agreements and divided by the number of total coding opportunities (agreements plus disagreements). We multiplied this number by 100% to get percentage of exact coding agreement between raters. Across the three transcripts IRR was high at 99% (MRS 1: 98%, MRS 2: 100%, and MRS 3: 100%). We discussed all disagreements and resolved them by consensus, with the third author reviewing all coded transcripts for agreement prior to data analysis.

#### Reflections

Following Braun and Clarke’s (2006) guidance for thematic analysis, the second author carefully analyzed and annotated participants’ written reflections before conducting open and process coding (Saladana, 2016). See the Supplementary files for an example of this coding process. Then, the second author developed initial themes, refining and analyzing codes to ensure themes were representative of the data. To ensure trustworthiness of the findings (Lincoln and Guba, 1985), all authors met to review the audit trail and memos made by the second author, as well as confirm that identified themes were supported by the data. Additionally, MRS transcripts for each participant group were reviewed during the coding process for triangulation purposes.

### Data analysis

To answer research question 1, we tallied verbal counts (Chi, 1997) for each code in our coding scheme for each participant, along with means, standard deviations and total percentages for each code across all participants. We include examples from each participant’s talk to provide further description of the utterances produced during each MRS. We also calculated ratios of participant and student (i.e., avatars) talk during MRS scenarios (i.e., total number of utterances and total number of words within those utterances produced by participants and avatars). For research question 2, we calculated average scores for participants’ self-efficacy for: (1) writing, (2) writing elements, and (3) writing instruction on the PTSWI. We then examined variability in participants’ instructional moves in relation to their beliefs and self-efficacy. For research question 3, we identified themes from participants’ reflections to provide further information on participant learning from MRS experiences.

## Results

In the sections that follow, we provide study results. For each research question, we first describe results across all three MRS scenarios, followed by results for each of the MRS and each of the five participants.

### Research question 1: instructional moves during MRS elementary writing conferences

#### Overall trends

Table 2 provides the counts and percentages of utterances across all MRS. The most common type of utterances participants made were those coded as *Manage* (31.7%), with about 7.60 (*SD* = 5.18) *Manage* utterances, on average, per MRS. *Manage* utterances included participants’ attempts to set expectations, clarify directions, and address avatar behavior. The next most frequent type of utterances were those used to instruct about the writing piece (*Instruct: Writing Piece,* 24.2%), with an average of 5.80 (*SD* = 3.96) of these responses per MRS. Instruction about the writing piece included utterances focused on editing, language, adding details, and actionable next steps for revising. Participants’ utterances were relatively evenly split across two additional categories: *Describe* (17.5%) and *Affirm* (20.0%). Utterances coded as *Describe* (*M* = 4.20, *SD* = 1.92) focused on describing the avatar’s writing piece, or questions to elicit description of the writing piece, while utterances coded as *Affirm* (*M* = 4.80, *SD* = 1.48) included encouragement and praise for the avatars and their writing. Participants rarely asked avatars to expand on the writing process or themselves as writers (i.e., *Expand*, 3.3%) and rarely instructed students about their development as writers (*Instruct: Writer*, 3.3%).

**Table 2:** Individual counts and overall percentages for participants’ utterances during MRS writing conferences.

Codes for utterances/Instructional moves						
	Describe	Expand	Affirm	Manage	Instruct	
					WP	WR
MRS 1						
Kim	4	0	3	9	0	0
Tanya	5	0	4	8	7	0
MRS 2						
Jackie	5	1	5	15	6	4
MRS 3						
Audrey	6	2	7	5	5	0
Sophie	1	1	5	1	11	0
Total count	21	4	24	38	29	4
Total %	17.5%	3.3%	20.0%	31.7%	24.2%	3.3%

WP, instruction on the writing piece; WR, instruction on development as a writer; MRS, mixed reality simulation.

##### MRS 1

In MRS 1, Kim and Tanya encountered a confident avatar who thought nothing should be changed in their writing. Both participants tended to follow the overall trends described above.

Kim and Tanya mostly talked to manage the avatars (i.e., Kim: 9 [56%] and Tanya 8 [33%] *Manage* utterances). Examples included Kim setting expectations at the beginning of the writing conference with, “Okay, so we are going to start off with our writing conference. I think I’m gonna start with Ava. And Dev and Jasmine, remember, I asked you guys to email each other your drafts last night. And what you guys are going to do is go over the other person’s draft and just see if you can add some comments or suggestions.” Tanya also spent time managing behavior. When an avatar interrupted her writing conference, Tanya said, “Oh one second. Jasmine, what do you want girly?.” The avatar responded by complimenting another avatar’s writing. Tanya replied by redirecting the interrupting student: “Aww that’s so nice, Jasmine. Thanks for letting Ava know… Can you save those comments for when you get a chance to give Ava comments on her paper?”

Kim and Tanya also described and elicited descriptions of the avatar’s writing, with 4 (25%) and 5 (21%) *Describe* utterances, respectively. For example, Kim said, “I just want to start off by saying that I’m so impressed by the way that you used structure throughout your writing. I saw that there was a very clear beginning, middle, and end…” Tanya focused on describing the avatar’s writing piece with, “I really like all the punctuation that you have used, I think that’s awesome. I can tell you paid attention during our punctuation lesson,” and later asked the avatar to read particular parts of their writing: “Can you read me the first sentence in your last paragraph?” Both participants had a similar number of utterances used to affirm avatars, such as when Kim said, “Ava, you are already off to an amazing start,” and Tanya repeatedly told the avatar “Awesome!” throughout the conference. Like the overall trends, Kim and Tanya did not make any utterances to expand upon the avatar’s writing process and neither participant instructed the avatar to focus on their development as a writer.

Kim and Tanya differed from the overall trends across MRS in that Tanya made 7 utterances related to instruction focused on the avatar’s writing piece (*Instruct: Writing Piece*, 29%), while Kim made none of these utterances. Tanya prompted the avatar to edit their writing for mistakes with plurals and then spent the remainder of the conference instructing on how to add details and description with utterances like, “Ava is there anything you think that we could add to the sentence to maybe make it a little bit more interesting?” After the avatar came up with ideas to add to their writing, Tanya reminded them to “make a little bitty note under your writing for now, so you know to go back later and add it in when we are writing our second drafts.”

##### MRS 2

Jackie encountered an avatar who was distracted by their classmates during the writing conference in MRS 2. Jackie followed overall trends for the types of utterances she produced during her MRS but was the only participant to talk about the avatar’s development as a writer.

Jackie’s most common utterances were those coded as *Manage* (*n* = 15, 42%). Like other participants, she spent time at the beginning of the conference setting expectations with statements like, “Okay, so today we are going to work on our writing. So Jasmine and Ava, I would like you two to please peer review each other’s work.” Later in the conference, she spent considerable time asking the other two avatars in the MRS to be quiet while she worked with the focal avatar: “Ava and Jasmine, would you guys mind, please reading each other’s works in your head?” and “Um, can you please be a little bit more quiet? It’s a little bit distracting.”

Like the overall trends, Jackie’s other utterances were split between *Describe* (*n* = 5, 14%) and *Affirm* (*n* = 5, 14%), with one utterance that expanded upon the writing process and the avatar as a writer (“Will you tell me how you are thinking about your writing today? How are you feeling about it?”). Jackie also focused instruction on the current writing piece (*n* = 6, 17%). After a discussion on adding detail to the avatar’s writing she said, “Do you think we can maybe add that into our sentence? Maybe his first wish is that he would have a blue mansion, and then you can add another sentence about how it’s on the beach and the mountains.”

Unlike any other participant, Jackie focused on development of the avatar as a writer with four statements (*Instruct: Writer,* 11%) focused beyond the current writing piece to prompt the avatar to think about audience (e.g., “We want to express in our writing how we see detail…We want them [the reader] to be able to close their eyes and be able to see exactly what you what you see”).

##### MRS 3

In MRS 3, Audrey and Sophie encountered a less-confident avatar who responded to teacher feedback as if it were criticism. Not only did Audrey and Sophie differ from each other in the utterances they made during the MRS, but they also differed from overall trends.

Unlike overall trends, Audrey’s utterances were most commonly coded as *Affirm* (*n* = 7, 28%) and *Describe* (*n* = 6, 24%); she had the greatest number of each of these utterances of all participants. To affirm and support the avatar, Audrey began the writing conference with statements like, “I really loved your writing piece” and “I think what you have so far is a really good start.” Later, when the avatar demonstrated they were not confident about their writing, Audrey said, “Oh, Jasmine, it was a lovely story” and “I happen to think you are very smart.” When describing the current writing piece, Audrey made statements about its structure, much like Kim had in an earlier MRS: “I really liked how you had a clear beginning, middle, and end.” Later, Audrey continued to describe the avatar’s writing with “I really liked that you gave Fred unlimited wishes…he got to wish for everything he wanted.”

Audrey had 5 each (20%) of *Manage* and *Instruct: Writing Piece* utterances. Like other participants, Audrey set expectations at the beginning of the writing conference with statements like “I’m going to start with Jasmine today, but Ava and Dev, I want you to pull out your writing rubrics that we have used in our class before, and I want you to go over each other’s writing and just give some comments.” Later, she checked for understanding with “Does that sound like a good idea?” When providing instruction focused on the current writing piece, Audrey made statements such as “We’re just going to add a little bit of detail to make it even better.” When the avatar came up with a detail to add, Audrey said, “That’s a great wish that we could add.” Like most participants, Audrey made no utterances focused on development of the avatar as a writer.

Although relatively small, Audrey had the most utterances of any participant coded as *Expand* (*n* = 2, 8%). Like Jackie, she checked in with the avatar, focusing on how they felt during the writing process with, “I was wondering, how did you feel when you are writing this piece? Did you feel good about it? Did you feel confident?” and later asked, “Did you enjoy writing that part of the story?”

Unlike Audrey and the rest of the participants, most of Sophie’s utterances were used to provide instruction related to the writing piece (*n* = 11, 58%). Sophie focused on supporting the avatar to add descriptive words to the text. She made comments focused on the writing piece such as, “So let us start with the first sentence, it says, Once upon a time there was a boy named Fred. Fred could be anyone. What did he look like to you?” When the avatar provided some description of their main character, Sophie continued instructing on the writing piece with, “A boring guy? Okay that’s a good descriptive word…Can you think of one more descriptive word?” She then had the avatar read the text, line-by-line, to add descriptive words. Sophie wrapped up instruction with reminders of next steps: “Why do not you write ‘add detail’ at the top of your paper and then you can go back and work on that for next time?” Like most participants, however, Sophie made no utterances focused on the avatar’s development as a writer.

Similar to Audrey, slightly more than one-quarter of Sophie’s utterances were coded as *Affirm* (*n* = 5, 26%). Sophie provided affirmation throughout the MRS with repeated use of “good” (e.g., “Boring is good” and “That’s a good detail”). She ended the conference with affirmation for all three avatars, “Great job today, Jasmine. I’m so proud of your work,” and “Thanks Ava and Dev for being so quiet.”

Sophie’s remaining utterances were evenly split (*n* = 1 each), between *Describe*, *Expand*, and *Manage*. Like other participants, Sophie described the use of details in the avatar’s writing and expanded upon the process by asking about how the avatar felt while writing. Her utterance coded as *Manage,* “Maybe we’ll save that idea for later,” was used to maintain focus and pacing in response to the avatar’s repeated answers of “ummmmmm” and “I do not know.”

##### Ratios of participant (teacher) to avatar (student) talk

Across all MRS, there was a total of 121 participant (i.e., teacher) utterances and 58 avatar (i.e., student) utterances; this equaled 2,427 participant words and 574 avatar words. Participants made more than twice as many utterances and said four times as many words as the avatars during the MRS writing conferences.

In MRS 1, Kim and Tanya made 40 utterances, while the avatars in their MRS contributed a total of 21 utterances; these utterances consisted of 953 participant and 251 avatar words. Thus, there were nearly twice as many participant utterances as avatar utterances, and Kim and Tanya spoke more than 3.5 times as many words as the avatars in their MRS.

In MRS 2, Jackie made 36 utterances and the avatars made 21 utterances. Jackie’s utterances equaled 611 words, while avatars produced 179 words. Although Jackie’s utterances were not double those of the avatars in her MRS, they did take up a majority (63%) of the talk during the writing conference. When examining words produced, Jackie produced more than three times as many words as the avatars in her MRS.

In MRS 3, Audrey and Sophie made 45 utterances while the avatars made 16 utterances; this equaled 863 participant words and 144 student words. Like the overall trend, Audrey and Sophie made more than two times as many utterances as the avatars in their MRS. Their word count was nearly six times that of the avatars in MRS 3.

### Research question 2: self-efficacy and variance with instructional moves during MRS elementary writing conferences

Table 3 shows each participant’s average score for self-efficacy for writing, self-efficacy for teaching writing elements, and self-efficacy for writing instruction (from the PTSWI), along with the type of utterances they most and least commonly made during the MRS. As shown, scores on the PTSWI were relatively similar across participants, with scores at or near 4, indicating strong, or high, self-efficacy in each area (Hodges et al., 2019).

**Table 3:** Averages for PTSWI with most and least common type of utterance in MRS.

	PTSWI			MRS	
	Writing	Elements	Instruction	Most	Least
Kim	4.00	4.00	4.54	Manage 56%	Expand, WP, WR 0%
Tanya	3.60	4.13	4.23	Manage 33%	Expand, WR 0%
Jackie	3.70	3.4	3.92	Manage 42%	Expand 3%
Audrey	3.60	3.87	3.85	Affirm 28%	WR 0%
Sophie	3.60	3.67	4.08	WP 58%	WR 0%

PTSWI, Pre-service Teachers’ Self-Efficacy for Writing Inventory; MRS, mixed-reality simulation; WP, instruction on the writing piece; WR, instruction on development as a writer.

Kim had the highest self-efficacy average overall (*M* = 4.18), and the highest averages in self-efficacy for writing (*M* = 4.00) and self-efficacy for writing instruction (*M* = 4.54). Kim also had most of her utterances (56%) coded as *Manage* (i.e., setting expectations, addressing behavior, clarifying directions) during her MRS; hers was the highest percentage of utterances coded as *Manage* across all participants. Kim was also the only participant with 0% for three categories: expanding on the writing process and the writer, instruction on the writing piece, and instruction in developing the student as a writer.

Tanya had the highest reported self-efficacy for teaching writing elements (*M* = 4.13). Tanya’s highest category of utterances were those used to manage behavior and expectations (33%) during the MRS. Twenty-nine percent of her utterances during the MRS focused on instruction on the writing piece (e.g., adding details, editing). However, 0% of Tanya’s utterances involved expansion on the writing process or the writer and 0% involved instruction designed to develop the avatar as a writer. Additionally, Tanya’s average self-efficacy for writing (*M* = 3.60) tied with Audrey and Sophie for the lowest of the participants.

Jackie had the lowest average self-efficacy for teaching writing elements (*M* = 3.40) of all participants. Although she mostly talked to manage the avatar’s behavior and expectations (42%), like Kim and Tanya, Jackie was also the only participant to focus on developing the avatar as a writer (11%), and her second most common type of utterance involved instruction focused on the writing piece (17%).

Audrey scored the lowest for self-efficacy for writing (*M* = 3.60, tied with Tanya and Sophie) and the lowest for self-efficacy for writing instruction (*M* = 3.85). Her most common type of utterance was used to affirm the avatar (*Affirm,* 28%; e.g., Great job!) and she focused 0% of her MRS on instruction on the writing piece.

As mentioned previously, Sophie tied for lowest self-efficacy for writing (*M* = 3.60), yet her MRS was predominated by utterances focused on instruction on the writing piece (58%), the most of any participant. Like Kim, Tanya, and Audrey, Sophie focused 0% of her MRS on developing the avatar as a writer.

### Research question 3: reflections on learning from the MRS

Through thematic analysis (Braun and Clarke, 2006), the researchers identified two overarching themes throughout participants’ reflections on their learning during the MRS. These themes are representative of how participants made sense of their experiences during their own interactions with the student avatars, as well as what they observed while watching their peers’ MRS experiences. We present each of these themes in more depth below.

#### Participants began to shift their thinking away from trying to plan “the perfect lesson” and recognize that adapting plans is an integral part of classroom teaching

Across their reflections, participants described a change in the way they conceptualized effective lesson planning. Some participants referenced the idea of trying to “plan a perfect lesson,” but their time in the MRS highlighted that a teacher cannot plan for every possible situation that may occur during instruction. For example, Jackie reflected, “Over the years we have learned how to make lesson plans and have taught them to our peers. We make them perfect and we meet time requirements…but we teach them to adults…No matter how perfect our lesson plan is on paper, it may not go that way in the classroom.” Although the participants were well-prepared and had included all parts of their lessons, once lessons were enacted with student avatars, they realized changes had to be made in response to students’ actions and needs.

One common reason for the changes was the constraint of time. Participants described pressure around attempting to complete a full writing conference in about 5 min and often found they had to cut parts of their lessons to complete the task in the time allotted. For example, Tanya described planning to go through a whole paragraph with her avatar but was only able to get through a single sentence. Similarly, Sophie acknowledged that although she and her team were able to complete what they had planned, it took much longer than they had anticipated: “While we were eventually able to get to this point and clarify this objective for the student to work on when they went back to their desk, it took a while to get there.” Participants found that completing their plans, once enacted with “real” students, took more time than they realized, and they had to make adjustments during conferences to achieve their intended outcomes.

Another common reason for adjustments made during the MRS related to the social and emotional needs of student avatars. Participants Kim and Tanya both highlighted the needs of the avatars, and that these needs should be accounted for when planning for future writing conferences. However, sometimes participants realized that student needs could not be planned for, and adjustments had to be made during the lesson to support students. For example, during Audrey and Sophie’s MRS, their avatar lacked confidence. They felt they could not move to the instruction portion of their lesson until they had sufficiently supported the avatar. In reflection, Audrey noted, “Since she was lacking confidence in her writing, we felt that it was necessary to spend more time encouraging her in her ideas and identity as a writer rather than making numerous edits to her composition.” These participants felt that to conduct a successful writing conference, the needs of the students must be addressed in the moment.

Across reflections, participants began to change their thinking around planning; recognizing that making changes during a lesson does not indicate lack of planning but is rather an important part of teaching. This idea was summarized by Sophie: “This experience was very enlightening because it showed me that while planning for a writing conference is a crucial element so that you are prepared to lead it as the teacher, things will more than likely turn out differently than you initially imagined.” Additionally, Kim stated, “I designed my plan according to a perfect classroom and perfect students. However, I now realize that this is not a logical way to create a plan after this experience.” These statements highlight participants’ recognition of the need for both detailed plans as well as the ability to adapt those plans to be able to conduct a successful conference.

#### Participants grappled with making in-the-moment decisions during their MRS experience but felt supported by their peers and were able to learn from observing one another

Although participants recognized the need for adjusting their lesson plans while in the MRS, they also highlighted the difficulties they faced in making in-the-moment decisions. Tanya described how she made an instructional move (i.e., allowing Ava to choose which paragraph to work on) that did not seem to engage the avatar. She stated, “This kinda threw me off because I expected her to pick, so then I had to quickly pick a paragraph to focus on.” This experience is also clear in Jackie’s reflection on an instructional move she made regarding student behavior:

One thing that I wish I handled better or differently was when Ava snapped at me. When I asked her to be quiet when she was distracting Dev, she responded with “whatever”. I was so shocked that she said that! I froze in the moment and didn't know what to say, so I said nothing at all…Was this the wrong thing to do? How would someone with more knowledge have handled this situation? This was something I wasn’t prepared for.

Jackie’s experience demonstrates how participants weighed possible options for instructional moves. In Jackie’s case, she felt she needed more knowledge or experience to make those decisions. Some participants also noted how they reflected on decision making after the fact, such as Kim who stated, “I was thinking of so many different ways that I could have approached the situation and how I could have corrected my mistakes in the moment. These quotations underscore how participants felt compelled during the MRS to make quick decisions in response to the avatars and reflected on the effectiveness of those decisions.

As participants reflected on their MRS, they highlighted the benefits of working with their peers, through both observing and supporting one another during instruction. Jackie noted she knew her peers were prepared to take over the simulation if she had to “tap out” (e.g., decide she no longer wanted to participate in the MRS). Similarly, Sophie mentioned how, during a pause, her peer group was integral in helping to decide which instructional move to make once the simulation resumed: “My peer group helped me decide to tell her that since we loved her writing so much, we wanted to hear more of it in order to get her motivated to add more descriptive detail to her writing.” In Kim’s reflection, she acknowledged that she struggled to get her avatar back on track and was grateful to step away from the MRS and allow her partner, Tanya, to try a new tactic. She stated, “Tanya taught me some ways that I can use to adapt my plan for difficult students.” Having peers available for support and problem-solving helped participants work through obstacles that arose during their MRS experiences.

Participants also acknowledged the influence of observing their peers’ MRS before their own and how those observations influenced their decision making. Audrey noted this was particularly helpful when it came to expectation setting, stating, “Especially in seeing how to set explicit behavior expectations for Dev and Ava, being able to watch other groups first helped us create a clear, explicit opening statement.” Several participants expressed this sentiment, particularly in reference to anticipating student behaviors. Jackie noted, “After seeing some of the student’s reactions, I knew we had to give them explicit instructions.” Moving forward, Sophie mentioned that learning from watching her peers may influence her future instructional moves during a writing conference: “It was so helpful having Audrey set the expectations at the beginning of the conference before I went it because I saw how explicit she was when giving directions to both Jasmine and the other two students, and I recognized how I wanted to be intentional about implementing that skill myself.”

## Discussion

In this study, we examined pre-service teachers’ talk during simulated writing conferences with elementary student avatars. We were interested in participants’ instructional moves (talk was used as a proxy for coding instructional moves) during each of three MRS writing conferences. We also examined if participants’ instructional moves varied in relation to their reported self-efficacy for: writing, teaching writing elements, and writing instruction. In response to our initial findings, we performed an additional analysis of participants’ reflections on learning from the MRS experience to provide further insight into the impact of this experience on pre-service teachers.

Based on our findings, we discuss implications for pre-service teacher preparation, including the need to provide opportunities for pre-service teachers to: (1) learn about and practice conducting effective writing instruction; (2) learn about important pedagogical choices (e.g., wait time, open-ended questioning); and (3) develop positive beliefs, self-efficacy, and motivation for writing.

### Instructional moves during MRS writing conferences

In line with our hypotheses for research question 1, pre-service teacher participants applied course-related learning during MRS writing conferences with an average of just under one-quarter of utterances focused on instruction related to developing the writing piece (24.2%; e.g., adding details, focusing on language used, steps for editing and revising) across all MRS and all participants. As we anticipated, however, management of avatars and the writing conference predominated MRS experiences (31.7%; e.g., setting expectations, behavior management). Furthermore, teacher talk predominated MRS writing experiences, with nearly twice as many utterances and four times as many words spoken by pre-service teacher participants as avatars across the three MRS.

Our findings support the need for continued focus on writing instruction for pre-service teachers. Although our participants indicated some grasp of how to design and implement targeted writing instruction during writing conferences, only one participant spent most of her instructional time focused on the writing piece and only one participant focused any instructional time on developing the student as a writer. These findings align with nationwide surveys of in-service teachers who report feeling unprepared to deliver writing instruction and spend little instructional time doing so (e.g., Kiuhara et al., 2009; Gilbert and Graham, 2010; Ray et al., 2016).

Pre-service teacher preparation programs have a responsibility for expanding literacy courses to include methods for providing effective writing instruction. Such efforts could capitalize on reading-writing connections, as reading predominates educator preparation coursework in the U.S. (Brenner and McQuirk, 2019), but writing, both learning to provide instruction on the component skills needed for writing *and* the composition process deserve a space in teacher preparation courses and applied experiences (Myers et al., 2016; Hawkins et al., 2022). Admittedly, our own pre-service preparation program has only one master’s level course devoted to K-12 writing instruction and our undergraduate courses mainly focus on how to provide reading instruction.

Changes to pre-service preparation may require shifts in state standards and federal policies (e.g., Reading First). Both tend to emphasize reading over writing and pre-service coursework may be reflective of these priorities which drive what is emphasized in our nation’s classrooms (Brenner and McQuirk, 2019). We strongly believe that with adequate pre-service preparation to teach writing and continued supports to provide effective writing instruction in the classroom (Wijekumar et al., 2019), U.S. teachers can reverse decades-long trends of students who do not have the writing skills needed for success in K-12 classrooms (e.g., Salahu-Din et al., 2008; National Center for Education Statistics, 2012; White et al., 2015) and beyond.

Because management and teacher talk predominated MRS writing conferences, pre-service preparation programs should also provide targeted instruction and practice opportunities around pedagogy and classroom management. For example, our pre-service participants appeared to need more preparation for how to quickly set expectations and use most of their time for instruction. Additionally, they frequently failed to provide wait time for students or to ask open-ended questions that allowed students to contribute ideas, with only 3.3% of overall utterances focused on prompts or questions for student avatars to expand on the writing process or their approach as a writer. The predominance of teacher talk may also indicate teachers’ use of talk as an attempt to control or manage the students and the MRS, or as Edwards and Furlong (1978) described, a way of “maintaining order” (p. 149). This further supports the need for pre-service teacher preparation programs to provide instruction on how to support and manage student engagement and behavior in the classroom so teachers can focus less on these aspects and spend more time providing high-quality academic-focused instruction.

### Self-efficacy and variance with instructional moves during MRS writing conferences

Our findings for research question 2 did not align with our hypotheses. Although we expected participants with higher self-efficacy would provide more writing instruction, this was not the case. In fact, Kim, the pre-service teacher participant with the highest self-efficacy average score and the highest self-efficacy average for writing and writing instruction, spent none of her MRS writing conference focused on writing instruction (*Instruct: Writing Piece,* 0%; *Instruct: Writer,* 0%). Conversely, Jackie, the participant with the lowest average self-efficacy score, was the only participant to implement instruction focused on developing the student as a writer (*Instruct: Writer,* 11%). Furthermore, Sophie, who tied Jackie and Audrey for the lowest average self-efficacy for writing score, was the only participant to spend a majority of her time focused on instruction on the writing piece (*Instruct: Writing Piece,* 58%).

These findings indicate a mismatch between pre-service teachers’ self-efficacy for writing and the writing instruction they enacted in the MRS. This supports the need for pre-service teacher preparation not only on how to provide effective writing instruction, but also preparation that facilitates self-efficacy and motivation for writing among pre-service teachers. Like Hodges et al. (2019), we believe pre-service preparation is the appropriate time to address self-efficacy, as changes in self-efficacy “take time and practice” and “reaching teachers who are still developing their beliefs about writing and writing instruction has the potential to proactively prepare teachers to more successfully integrate writing into their future classrooms rather than to reactively try to change entrenched behaviors” (p. 3–4).

### Reflections on learning from the MRS

Participants were asked to reflect on how they adapted their plans during the simulation, what factors may have influenced those adaptations, and what they would do the same or differently in a future writing conference. Although we were unsure of specific areas they would focus on in their reflections, we hoped participants would reflect on both their strengths and areas of need related to writing instruction and lesson planning. However, reflections revealed important learnings beyond just how participants chose to adapt and deliver their lesson plans. The first overarching theme from participants’ responses showed reflections on adapting lesson plans in response to the realities of implementing a writing lesson plan with student avatars. The second theme involved reflections on the difficulty of in-the-moment decision making during instruction and what was learned from peers during the MRS experience.

Participants’ reflections supported the use of teaching simulations in pre-service teacher preparation programs. Through the MRS writing conferences, our participants were able to better understand and practice adapting to the realities of an actual classroom, which they could not do through lesson planning alone or through teaching to peers. Participants had valuable take-aways from the MRS experience related to time and behavior management and making in-the-moment decisions to adjust plans. This type of learning would not have been possible without the simulated teaching environment that promoted growth in understanding of lesson planning, but perhaps more importantly, provided the opportunity to actually experience how to make on-the-fly adjustments to lesson plans based on some of the demands and needs one would have in an actual classroom.

We believe that applied experiences focused on writing will allow pre-service teachers to better understand their own writing instruction abilities, so they enter the classroom with a more precise understanding of the challenges they may encounter with writing instruction and management of the learning environment. Pajares (1996) described this revision, or better understanding of one’s self-efficacy with children and adolescents, as a “recalibration” that helps students “better understand what they know and do not know so that they may more effectively deploy appropriate cognitive strategies as they perform a task,” (p. 355). In the same way, we believe teachers who enter the classroom with a better understanding of their own skills and a better understanding of instruction, pedagogy, and classroom management, will be more successful, and teachers who are more successful are more likely to be motivated to remain in the profession (Schunk, 1995; Lauermann et al., 2017).

Learning from peers in the MRS writing conferences was also powerful and showed in our results and reflections. Sophie, the final participant in the MRS writing conferences, had the fewest (only 1) utterances related to management of the avatar and writing conference, while the first three participants’ MRS writing conferences had *Manage* as their most common type of utterance. Furthermore, Sophie’s conference predominantly involved instruction on the writing piece (58%) and she noted in her reflection that her partner (who taught in the conference directly before her) had already spent time setting expectations and providing directions for what the avatars should do; thus, she could focus on instruction. Although our participants only completed one MRS experience, we hope that providing multiple opportunities for approximations of teaching practice through simulation, pre-service teachers will increase their own self-efficacy as they became more and more successful and as they learn from peers who are successful (Schunk, 1995; Usher and Pajares, 2008). From this, teachers with higher self-efficacy and greater motivation to teach writing will likely provide more and better writing instruction as well as have students who demonstrate higher writing performance (e.g., De Smedt et al., 2016; Graham et al., 2022).

Participants’ reflections on learning from each other during the MRS experience support continued use of simulations in pre-service teacher preparation. Participants reflected on what they learned from observing others and what they learned from having a group of peers with whom they could confer and strategize with to address events they had not initially planned for. This type of learning would not be possible without the opportunity to implement instruction with student avatars, who behaved in ways similar to actual students, and without the option to pause, confer with peers and the professor, and restart instruction that Mursion afforded (Dieker et al., 2014). We feel strongly that teaching simulations, via Mursion and other related technologies, should be an integral part of pre-service teacher preparation, as they provide a link between coursework and applied practice in field placements, a sort of interim space to experiment with ideas and instruction with lower stakes than an actual classroom full of students (Bradley and Kendall, 2014).

### Limitations and future research

We recognize that our work is both exploratory and descriptive. Thus, we can describe participants’ utterances, self-efficacy, and reflections but do not draw causal connections between the MRS writing conference and participants’ behaviors or performance. This work is new, and we are designing next iterations of MRS writing conferences moving forward. We hope other researchers will begin examining the impact of MRS in pre-service preparation to teach writing, as explorations of the impact of MRS have predominantly been conducted in other subjects (e.g., Grant and Ferguson, 2021). Because this work is emergent, future studies should continue to employ mixed methods to better understand what works and under which conditions, and researchers should aim to draw causal connections between MRS and pre-service teacher outcomes, both in their preparation programs and in their future classrooms.

We also acknowledge that our design and assessment choices impacted our findings. We chose to develop three MRS scenarios to avoid participants being overly influenced by the instructional moves of the groups before them. However, it is possible that each of our three scenarios may have encouraged different types of instructional moves from our participants. We further allowed for collaborative planning among group members prior to MRS; this, along with group composition, could have impacted participants’ instructional moves. We also recognize the limitations of using a single instrument to measure participants’ self-efficacy for writing instruction through self-report. Future research should explore how MRS scenario contexts, collaborative planning, and group composition could impact instructional moves during MRS writing conferences. Our findings from participants’ reflections also provide reasons to further explore how participants benefit from observing peers in MRS scenarios before them and the types of learning that occurs between peers and between MRS sessions. Future research using multiple measures of self-efficacy for writing is important as well and research to examine if self-efficacy changes because of participating in the MRS.

We further understand that MRS alone, conducted once in a pre-service preparation program, is insufficient to cause lasting change in participants’ teaching practices. Coaching during teaching simulations has been shown to be an important addition to MRS experiences (e.g., Cohen et al., 2020). In addition to further studies on coaching and feedback that is most beneficial for pre-service teachers in MRS experiences, we hope future research will assess the impact of multiple opportunities for pre-service teachers to participate in MRS experiences throughout their preparation programs. We believe that through multiple opportunities to approximate teaching practices in simulated environments, pre-service teachers will be more likely to develop instructional and pedagogical skills that could have lasting impacts on their future teaching and their future students (Darling-Hammond, 2006).

## Conclusion

We advocate for changes to teacher preparation programs that increase emphasis on the teaching of writing and support teachers’ self-efficacy and motivation for teaching writing. Such changes can have powerful impacts on the readiness of teachers as they first enter the field, their determination and persistence in the face of difficulties, their desire to remain in the profession, and their impact on students’ learning (Schunk, 1995; Yost, 2006; Graham et al., 2022). Our findings support the use of practice-based teaching opportunities, like MRS, that allow pre-service teachers to hone their instructional and pedagogical skills, and perhaps their self-efficacy too, in a space where they can take chances, get feedback, and learn from their peers and professors, without the multiple demands they will juggle in an actual classroom. Such opportunities present an invaluable avenue for future research and for the future of teacher preparation.

## Data availability statement

The original contributions presented in the study are included in the article/Supplementary material, further inquiries can be directed to the corresponding author.

## Ethics statement

The studies involving humans were approved by SMU Institutional Research Board (IRB). The studies were conducted in accordance with the local legislation and institutional requirements. The participants provided their written informed consent to participate in this study. Written informed consent was obtained from the individual(s) for the publication of any potentially identifiable images or data included in this article.

## Author contributions

AGR, MY, and DG contributed to study design and data analysis. AGR took primary responsibility for writing the manuscript, with writing support from MY and editing support from DG. All authors contributed to the article and approved the submitted version.

## Conflict of interest

The authors declare that the research was conducted in the absence of any commercial or financial relationships that could be construed as a potential conflict of interest.

## Publisher’s note

All claims expressed in this article are solely those of the authors and do not necessarily represent those of their affiliated organizations, or those of the publisher, the editors and the reviewers. Any product that may be evaluated in this article, or claim that may be made by its manufacturer, is not guaranteed or endorsed by the publisher.
