# Supplementary material for: Exploring relationships between pre-service teachers’ self-efficacy for writing and instruction provided in simulated elementary writing conferences
Source: Front Psychol. 2023 Sep 27;14:1214086. doi: 10.3389/fpsyg.2023.1214086 (PMC10565953; doi:10.3389/fpsyg.2023.1214086)
Supplement: Supplementary file 1 [file Data_Sheet_1.pdf]

Coding Scheme for Participants' Utterances During MRS Writing Conferences

Code	Describe	Expand	Affirm	Manage	Instruct: WP	Instruct: WR
Description	Describing the current writing piece	Expansion on the writing process and writer	Positive support and/or encouragement	Management of the conference and students	Developing the current writing piece	Developing the student as a writer
Examples	What is your writing piece about?<br><br>Who is the main character?<br><br>I like how you used . . .<br><br>Can you read me ____ sentence?<br><br>Where do you want to start?<br><br>What part of your writing should we work on?	How did you feel when writing this?<br><br>What are you working on as a writer?<br><br>Tell me about how you felt when writing this.	Yeah<br><br>Right<br><br>Great idea<br><br>I love your writing	Referencing previous lessons/ activating background knowledge<br><br>Setting expectations for what is to come during the conference<br><br>Addressing behavior (talking, phone use)<br><br>Clarifying directions and/or instructions	Writing skills (editing, handwriting)<br><br>Language (adjectives, nouns)<br><br>Adding details<br><br>Actionable, concrete next steps to revise/edit writing	Thinking about/ addressing the audience (writing with a reader's eye)<br><br>Author's purpose<br><br>Feedback about students' writing choices and style

WP = writing piece; WR = writer

Example Coding Procedures

Code	Definition	Example
The perfect lesson	Refers to how planned materials were not conducive to MRS experience.	"I designed my plan according to a perfect classroom and perfect students. However, I now realize that this is not a logical way to create a plan after this experience."
In-the-moment decision making	Refers to choices made by preservice teacher during the MRS during situations that arose which the preservice teachers did not plan for.	"I did decide to change my discussion questions based on what Ava and I were discussing in that moment."<br><br>"For example, I tried to give Ava a choice of which paragraph she wanted to work on but she said that she didn't really care. This kinda threw me off because I expected her to pick so then I had to quickly pick a paragraph to focus on."
Time constraints	Refers to adaptations made during the MRS due to time limitations, or how certain aspects of the lesson took longer than anticipated.	"While we were eventually able to get to this point and clarify this objective for the student to work on when they went back to their desk, it took a while to get there."<br><br>"In my opening of the writer's workshop, I spent more time than I had intended asking questions about things Jasmine liked in her writing and also pointing out components of her draft which I felt were strong."
Anticipating student behavior	Refers to observations preservice teachers made from previous groups about students' responses, and the ways in which they adapted their approach to account for these behaviors.	"After watching the groups before us, we realized that we needed to come up with a plan for the other two students while we worked with the one child."<br><br>"Another thing that I learned by watching the other groups was that I didn't want to say something like 'I think we should add more details in order to make our writing more interesting'. Almost every time this was said, the student would reply 'so you don't think my work is already interesting?'."
